# Peroxisome Proliferator-Activated Receptor-**γ** Ligands Alter Breast Cancer Cell Motility through Modulation of the Plasminogen Activator System

**DOI:** 10.1155/2011/594258

**Published:** 2011-10-29

**Authors:** Jennifer C. Carter, Frank C. Church

**Affiliations:** ^1^Department of Pathology and Laboratory Medicine, School of Medicine, The University of North Carolina at Chapel Hill, Chapel Hill, NC 27599, USA; ^2^Department of Pathology, Vanderbilt University, 161 21st Ave, C-3314 MCN, Nashville, TN 37232-2561, USA; ^3^Department of Pharmacology, School of Medicine, The University of North Carolina at Chapel Hill, Chapel Hill, NC 27599, USA; ^4^Division of Hematology-Oncology/Medicine, School of Medicine, University of North Carolina at Chapel Hill, Campus Box 7035, Chapel Hill, NC 27599-7525, USA

## Abstract

We investigated peroxisome proliferator-activated receptor-**γ** (PPAR-**γ**) ligands effect on cell motility and the plasminogen activator system using normal MCF-10A and malignant MCF-10CA1 cell lines. Ciglitazone reduced both wound-induced migration and chemotaxis. However, the effect was not reversed with pretreatment of cells with the PPAR-**γ**-specific antagonist GW9662. Immunoblot analysis of conditioned media showed ciglitazone decreased plasminogen activator inhibitor-1 (PAI-1) in both cell lines; this effect was also unaltered by PPAR-**γ** antagonism. Alternatively, treatment with the **ω**-6 fatty acid arachidonic acid (ArA), but not the **ω**-3 fatty acid docosahexanoic acid, increased both MCF-10A cell migration and cell surface uPA activity. Pretreatment with a PPAR-**γ** antagonist reversed these effects, suggesting that ArA mediates its effect on cell motility and uPA activity through PPAR-**γ** activation. Collectively, the data suggest PPAR-**γ** ligands have a differential effect on normal and malignant cell migration and the plasminogen activation system, resulting from PPAR-**γ**-dependent and PPAR-**γ**-independent effects.

## 1. Introduction

A function of any tumor cell that allows for propagation of diseased cells is the ability for that tumor cell to invade the surrounding tissue. One family of proteins involved in this pathological process is the plasminogen activator (PA) system [1, 2]. The PA system includes the urokinase-type plasminogen activator (uPA). uPA is most active when bound to its cell surface urokinase receptor, uPAR. In addition to the role of the uPA/uPAR complex in the degradation of the ECM, this complex plays a role in cell adhesion. uPAR is able to engage cell surface integrins, allowing for attachment of cells expressing the uPA/uPAR complex to other surrounding cells. Another key component is plasminogen activator inhibitor-1 (PAI-1), the physiological inhibitor of uPA activity [1, 2]. PAI-1 binds uPA bound to the cell surface, forming a PAI-1/uPA/uPAR complex that is then recognized by the scavenger protein low-density lipoprotein receptor-related protein (LRP), which internalizes the tertiary complex [3, 4]. Paradoxically, elevated levels of PAI-1 in breast cancer patients are associated with decreased patient survival [5]. 

Peroxisome proliferator-activated receptor-gamma (PPAR-*γ*) is a transcription factor that is considered the master regulator of adipogenesis [6, 7]. However, PPAR-*γ* has been found in numerous cell lines, including endothelial cells [8, 9], normal and malignant prostate epithelium [10, 11], and normal and malignant breast epithelium [12]. PPAR-*γ* is a ligand-activated nuclear transcription factor and the target of the thiazolidinedione (TZD) class of insulin sensitizing drugs [6, 13]. Drugs in this family bind PPAR-*γ*, resulting in the activation of the PPAR-*γ*/retinoid X receptor (RXR) heterodimer. PPAR-*γ* then binds the PPAR response element (PPRE) in the promoter of target genes, recruits coactivators, and then the gene is transcribed. In addition to TZD drugs, PPAR-*γ* has been shown to be activated by the naturally occurring 15-deoxy-Δ^12,14^-prostaglandin J2 (15d-PGJ2) [14]. Although 15d-PGJ2 is a potent agonist for PPAR-*γ* *in vitro*, there is data suggesting 15d-PGJ2 is not found at a high enough concentration to act as an *in vivo* ligand for PPAR-*γ* [15]. 

In addition to the TZD class of drugs and 15d-PGJ2, PPAR-*γ* has also been shown to be activated by a number of dietary fatty acids, specifically omega-3 (*ω*-3) and omega-6 (*ω*-6) fatty acids. A diet high in fat is associated with the development of a number of diseases, including cardiovascular disease, type 2 diabetes mellitus, and a variety of cancers. Dietary fat intake has been linked to prostate cancer risk [16], colon cancer [17–19], and breast cancer [20]. Thoennes, et al., showed differential transcriptional activity by PPAR-*γ* following treatment of MCF-7 cells with *ω*-3 and *ω*-6 fatty acids [21]. Treatment with *ω*-3 fatty acids inhibited levels of PPAR-*γ* activation, while *ω*-6 fatty acids increase PPAR-*γ* activity over control [21]. 

The goal of this study was to investigate the effect of PPAR-*γ* ligands on breast cancer cell motility and the plasminogen activator system. The TZD ciglitazone decreased cell motility, independent of PPAR-*γ*. PAI-1 levels were lower following ciglitazone treatment. The naturally occurring PPAR*γ* ligand 15d-PGJ2 also reduced wound-induced cell migration. Interestingly, treatment with the *ω*-6 fatty acid arachidonic acid (ArA) increased cell motility, while the *ω*-3 fatty acid docosahexanoic acid (DhA) had no significant effect. Our collective results suggest that the PPAR-*γ* ligand ciglitazone decreases cell motility, in a PPAR-*γ* independent manner, potentially though the down-regulation of PAI-1; alternatively, the PPAR-*γ* ligand ArA promotes migration in a PPAR-*γ* dependent manner that increases uPA.

## 2. Materials and Methods

### 2.1. Cell Culture

MCF-10A and MCF-10CA1 cells (obtained from Dr. F. Miller, Wayne State University, Detroit, Mich, USA) were cultured as previously described [22, 23]. All cell lines were cultured in DMEM:F12 (GIBCO, Invitrogen, Carlsbad, Calif, USA) containing 5% horse serum (HyClone, Logan, UT), 1% PSF (GIBCO, Invitrogen, Carlsbad, CA), 20 mg/mL EGF (Invitrogen, Carlsbad, Calif, USA), 50 ng/mL hydrocortisone, 100 ng/mL cholera toxin (CalBiochem, San Diego, Calif, USA), and 10 mg/mL insulin (GIBCO, Invitrogen, Carlsbad, Calif, USA). Cells were grown in a humidified atmosphere of 5% CO_2_ at 37°C as previously described [1].

### 2.2. *In Vitro* Wound Healing Assay

Cells were plated at 1.0 × 10^5^ cells per well in a 12-well tissue culture treated plate as detailed previously [24, 25]. At confluence, cells were serum-starved overnight. Cells were then scratched with the tip of a sterile yellow pipet tip and serum-free media containing various concentration of 15d-PGJ2 (Calbiochem, San Diego, Calif, USA) or ciglitazone ranging to 10 *μ*M (Cayman Chemical, Ann Arbor, Mich, USA) from ethanol stocks were added to each well. Migration was monitored at 0, 6, and 12-hours using a Kodak MDS290 camera. Wound closure was quantified by measuring distance as pixels between each leading edge of the wound (10 lines/wound) at each time point using the measuring tool in Adobe Photoshop, with a grid superimposed on image to guide measurements.

### 2.3. Modified-Boyden Chamber Assay

 Following serum starvation, cells were treated with PPAR-*γ* ligands ranging to 10 *μ*M of ciglitazone, ArA (ArA-sodium salt, Sigma, St. Louis, Mo, USA), or DhA (DhA-sodium salt, Sigma, St. Louis, Mo. USA) in serum-free media for 24 hours. Lower wells of chamber contained DMEM:F12 plus 1 mg/mL fatty-acid-free bovine serum albumin (BSA, Sigma, St. Louis, Mo, USA) with or without 5 ng/mL EGF (Invitrogen, Carlsbad, Calif, USA). Cells (1 × 10^5^) were plated in upper wells in DMEM:F12 containing 1 mg/ml fatty-acid free BSA, above a collagen IV coated, 10 mm porated membrane. Chambers were incubated at 37°C for 6-hours in a humidified atmosphere. Cells were fixed and stained with Diff-Quick (Dade-Behring, Newark, DE). Cells that migrated to the undersurface of the membrane were examined microscopically at 200x magnification. Each condition was done in triplicate, with 4 fields counted per well [1]. In experiments with GW9662, serum-starved cells were pretreated for 30 minutes with GW9662 (5 *μ*M) (Calbiochem, San Diego, Calif, USA), then ciglitazone or fatty acid treatment was added to cells for 24-hours. GW9662 is an irreversible PPAR-*γ* antagonist and it was used at a concentration where it is selective for PPAR-*γ* in cells [26, 27].

### 2.4. Cell Viability Assay

Cells were plated at 1.0 × 10^4^ cells per well in a 96-well tissue culture plate. Confluent cells were serum-starved 24 hours, then MTT (3-(4,5-dimethylthiazol-2-yl)-2,5-diphenyltetrazolium bromide) reagent was added to cells and incubated at 37°C for 3-hours. Supernatant was removed and cells were washed in PBS. DMSO was added to cells and incubated at 37°C for 30 minutes. Absorbance was measured (Abs = 595 nm) on a SpectraMax microplate reader (Molecular Devices, Sunnyvale, Calif, USA).

### 2.5. Immunoblot Analysis

Conditioned media from treated cells was collected and concentrated with centrifugal concentrators (Amicon Ultracel 30 kD, Millipore, Billerica, Mass, USA). Protein concentration was determined using BioRad Protein DC assay (BioRad, Hercules, Calif, USA). Proteins were separated by SDS-PAGE in 10% polyacrylamide and electrotransferred to PVDF membrane. Phosphate buffered saline/0.1% Tween-20 (PBS/Tween) buffer was used in all steps of immunoblot analysis. Each step was preceded by three 9-minute washes at room temperature. Nonspecific binding was blocked by 5% nonfat dry milk for 30 minutes at room temperature. Membrane was incubated at 4°C overnight with primary antibody diluted 1 : 1000 (unless otherwise noted) in 1% nonfat dry milk. Membrane was exposed for 1-hour at room temperature to horseradish peroxidase conjugated secondary antibody diluted 1 : 5000 in 1% nonfat dry milk in PBS/Tween. Membrane was exposed to luminal substrate for 1 minute, covered in plastic wrap then exposed to X-ray film. Primary antibodies were: rabbit antihuman PAI-1 (1 : 2000 dilution) (Molecular innovations, Novi, MI) and rabbit anti-human uPA (no. 389, American Diagnostica, Stamford, Conn, USA) as described previously [1].

### 2.6. Indirect Cell-Surface Associated UPA Activity Assay

MCF-10A and MCF-10CA1 cells (1 × 10^5^) were plated in a 96-well plate [1]. Following 24-hour serum starvation, cells were pretreated with PPAR-*γ* antagonist GW9662 or vehicle control for 30 minutes at 37°C. Cells were then treated with various concentrations (up to 10 *μ*M) of ciglitazone or arachidonic acid for 24 hours at 37°C. After treatment, cells were washed with PBS and plasminogen was added to cells and incubated at room temperature. The supernatant was removed and added to another 96-well plate containing buffer amiloride to inhibit any residual uPA activity. Chromogenic substrate is then added to the well and hydrolyzed by plasmin generated by plasminogen cleaved by uPA on the cell surface. Rate of chromogenic substrate cleavage by plasmin was measured at 405 nm for 90 minutes.

## 3. Results

### 3.1. Plasminogen Activator, PPAR-*γ*, and RXR in MCF-10A and MCF-10CA1 Cells

As previously reported, MCF-10A cells express less uPA and uPAR but more PAI-1 than MCF-1CA1 breast cancer cells [1]. Both cell lines express PPAR-*γ* and RXRs (data not included). Based on these findings, we performed a study with some PPAR-*γ* ligands on uPA/PAI-1-mediated cell migration processes comparing near normal MCF-10A cells to oncogenic Ras-transformed metastatic MCF-10CA1 cells.

### 3.2. PPAR-*γ* Ligands Decrease In Vitro Wound Closure

**Figure 1:**
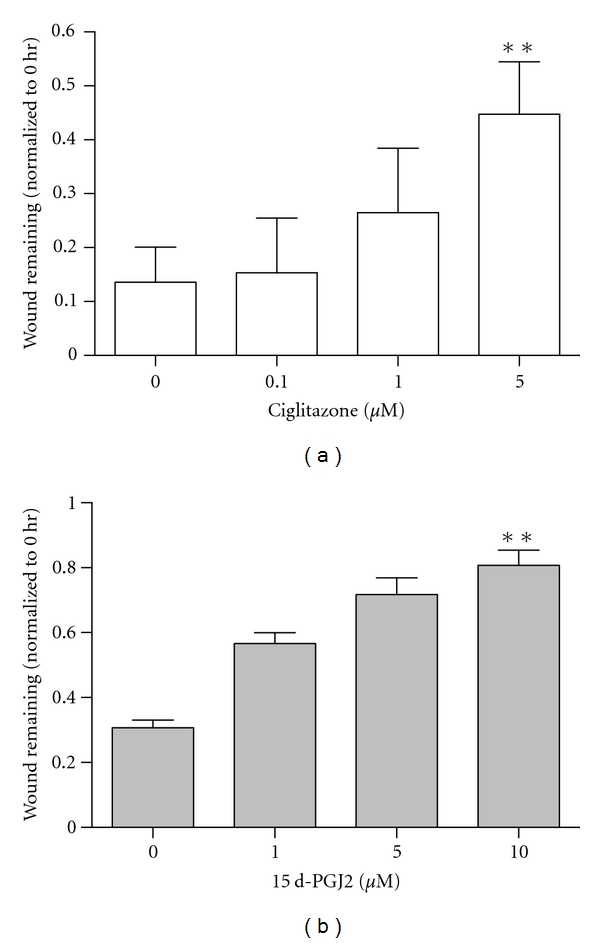
PPAR-*γ* ligands decrease wound closure in MCF-10A cells. Ciglitazone (a) and 15d-PGJ2 (b) were added to cells, and wound-induced closure measured as detailed under Section 2. Data shown are the 12-hour time point + SD (*n* = 3)  **P < 0.01.

Ciglitazone decreased wound closure dose dependently (Figure 1(a)), with 5 *μ*M ciglitazone reducing cell closure by 39% compared to no ciglitazone. 15d-PGJ2 also decreased cell closure dose dependently, with 10 *μ*M 15d-PGJ2 reduced cell closure by 50% compared to no 15d-PGJ2 (Figure 1(b)). These results show that PPAR-*γ* ligands decrease wound closure of MCF-10A cells, and they further support the literature that PPAR-*γ* activation inhibits migration of cancer cells *in vitro*.

### 3.3. Ciglitazone Treatment Decreases Chemotaxis, Decreases PAI-1 Expression, but Increases uPA Activity

**Figure 2:**
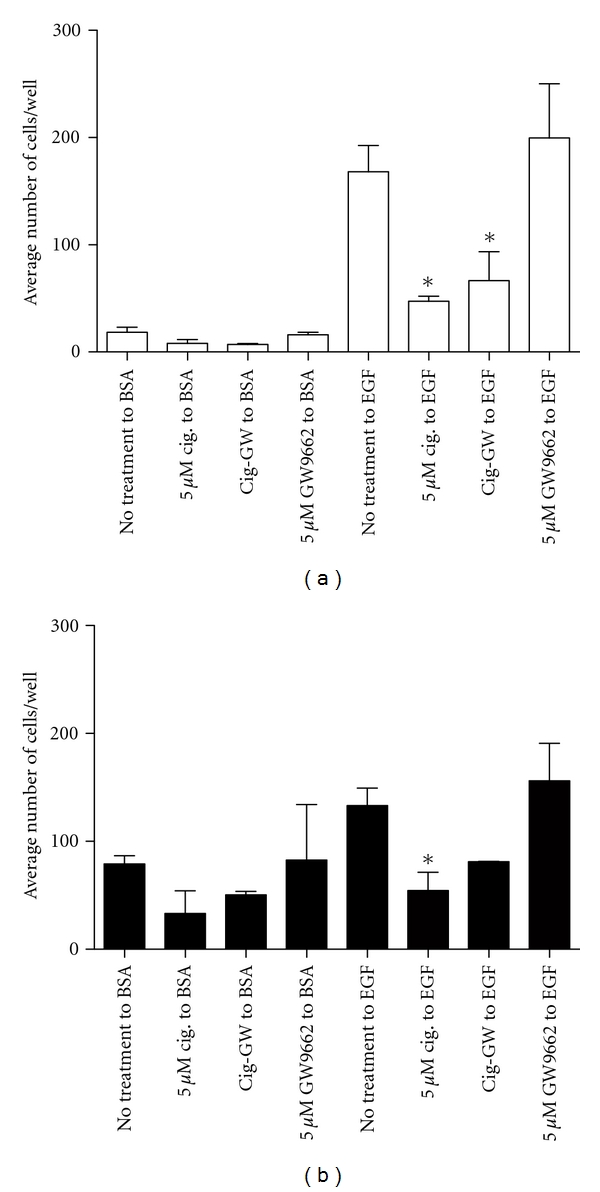
Ciglitazone decreases chemotaxis in MCF-10A (a) and MCF-10CA1 cells (b). Chemotaxis to either BSA (1 mg/mL fatty-acid free BSA) or to EGF (5 ng/mL EGF in 1 mg/mL fatty acid free BSA) was performed as detailed under Section 2. Each condition was done in triplicate. Values represent total number of cells per well + SD (*n* = 3)*P < 0.05.

Ciglitazone decreased cell chemotaxis to EGF in a dose-dependent manner (Figure 2). To determine if these effects were mediated by PPAR-*γ*, we pretreated the cells with the PPAR-*γ* specific antagonist GW9662. Interestingly, blocking PPAR-*γ* activation with GW9662 (5 *μ*M) pretreatment did not reverse the effect of ciglitazone (5 *μ*M) in either cell line. Control experiments with 5 *μ*M GW9662 showed neither detrimental effect on cell viability nor changes in cell motility. The data suggest ciglitazone is working in a PPAR-*γ* independent manner to reduce cell migration. The effect of ciglitazone on cell viability was then determined by MTT assay. Treatment of MCF-10A and MCF-10CA1 cells with 5 *μ*M ciglitazone partially reduced cell viability (Abs 595 nm of cells with no and 5 *μ*M ciglitazone was 0.331 ±  .014 and 0.292 ±  .003 for MCF-10A cells and 0.304 ±  .006 and 0.279 ±  .002 for MCF-10CA1 cells, resp.). There was a substantial loss of cell viability at 10 *μ*M ciglitazone for both cell lines; thus, all further experiments used 5 *μ*M ciglitazone. In additional control experiments, there was no loss of cell viability with the PPAR-*γ* ligands 15d-PGJ2 or ArA when tested up to 10 *μ*M (data not included). These results imply that the effect of ciglitazone in MCF-10A and MCF-10CA1 cell motility is not due to a substantial reduction in cell viability. 

**Figure 3:**
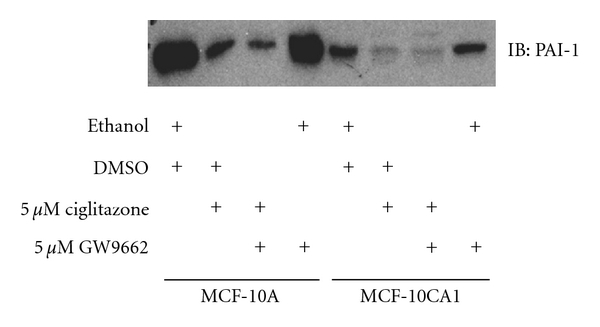
Ciglitazone decreases PAI-1 expression in MCF-10A and MCF-10CA1 cells and is not reversed by PPAR-*γ* antagonist pretreatment. Conditioned media from cells treated with ciglitazone (0–5 *μ*M), in the absence and presence of 5 *μ*M GW9662, was concentrated and 10 *μ*g total protein was separated by SDS-PAGE on a 10% polyacrylamide gel. Proteins were then transferred to PVDF membrane and probed for PAI-1 (rabbit antihuman PAI-1 antiserum) as detailed under Section 2.

In both MCF-10A and MCF-10CA1 cell lines, ciglitazone treatment resulted in decreased PAI-1 protein expression (Figure 3). To determine if this decrease in PAI-1 expression was mediated by PPAR-*γ*, we pretreated with GW9662 prior to ciglitazone treatment. We did not see a reversal of ciglitazone-mediated reduction in PAI-1 expression (Figure 3) suggesting ciglitazone is affecting PAI-1 levels independently of PPAR-*γ*. 

**Figure 4:**
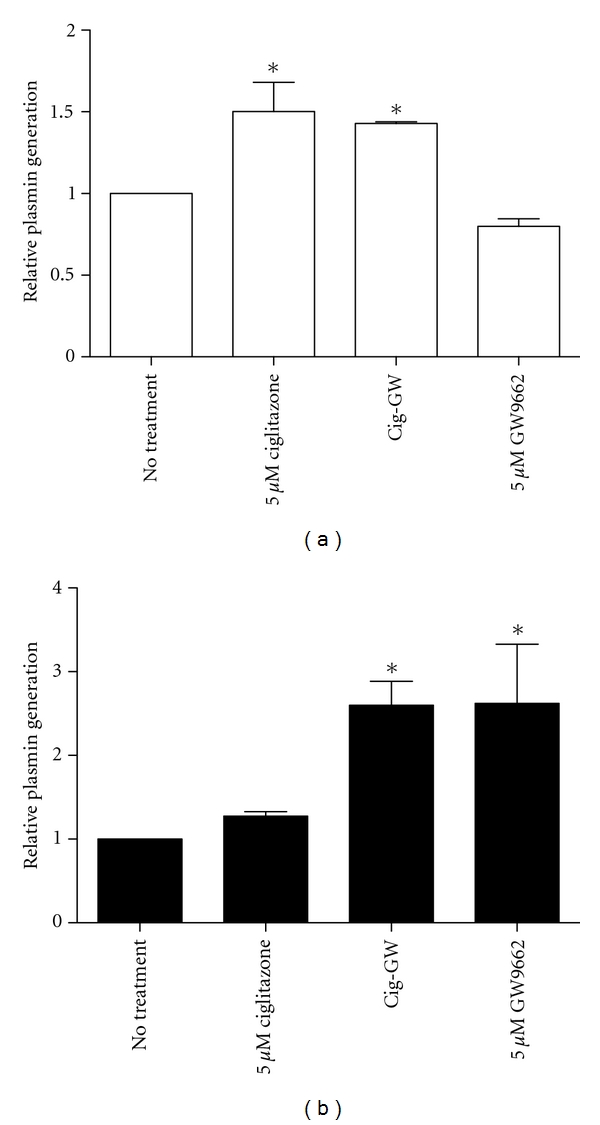
Ciglitazone treatment increases uPA activity in MCF-10A cells (a) but not in MCF-10CA1 cells (b). Following 24-hour treatment with ciglitazone (0–5 *μ*M), in the absence and presence of 5 *μ*M GW9662, media was removed from cells, washed in 1x PBS and plasminogen was then added, after 30-minutes at room temperature, cell supernatant was transferred to wells containing plasmin chromogenic substrate (S-2251, Chromogenix). Kinetics were read at 405 nm for 1.5-hours at 37°C. Values represent average *V*_max_ at Ab 405 nm, normalized to no treatment control, each condition done in triplicate (*n* = 3)*P < 0.05.

In MCF-10A cells, ciglitazone treatment alone or in conjunction with GW9662 pretreatment increases uPA activity on the cells surface (Figure 4(a)). Ciglitazone treatment in MCF-10CA1 cells did not significantly alter uPA activity although it seems GW9662 treatment in these cells results in more plasmin generation (Figure 4(b)).

### 3.4. Arachidonic Acid Increases Both MCF-10A Cell Motility and uPA Activity

**Figure 5:**
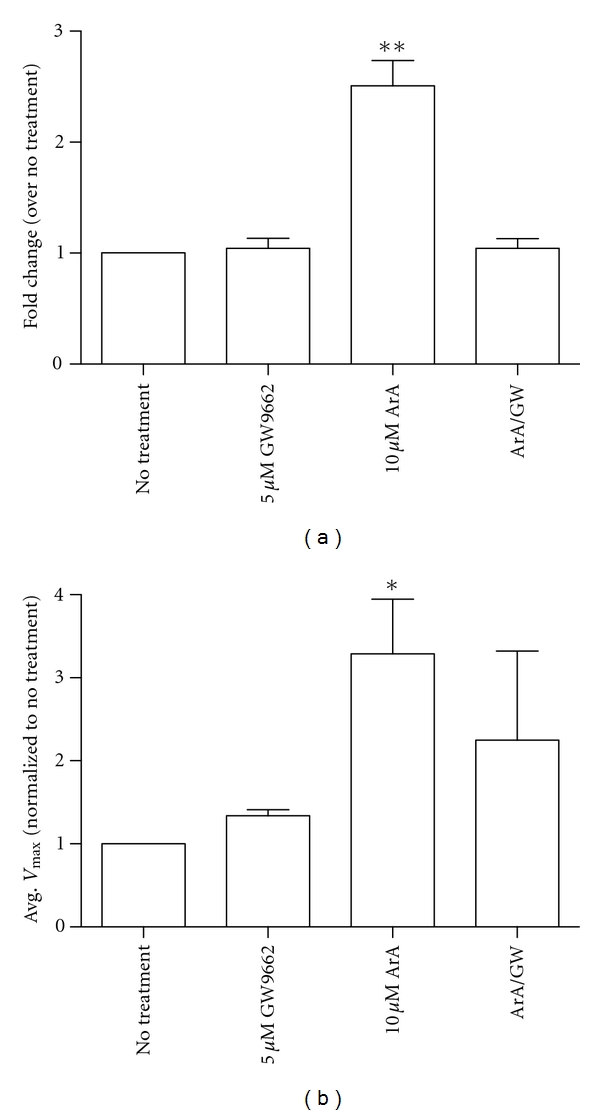
Arachidonic acid treatment increases MCF-10A cell motility (a) and uPA activity (b). MCF-10A cells were treated for 24-hours in serum-free media containing 10 *μ*M ArA or vehicle control (absolute ethanol in DMEM:F12 containing 1 mg/mL fatty acid-free BSA) following a 30 min pretreatment with GW9662 (5 *μ*M) or vehicle (DMSO) as detailed under Section 2. Values represent average number of cells chemotaxing to EGF/well + SD. *n* = 3  **P < 0.01. Separately, after receiving the same treatment as above, cells were washed in PBS, then incubated in buffer containing plasminogen. Supernatant from cells was transferred to a new well containing buffer, amiloride and plasmin substrate. Values represent average *V*_max_ (Abs 405 nm) + SD (*n* = 3)*P < 0.05.

With 10 *μ*M ArA, we see a significant increase in cell motility compared to control cells (Figure 5(a)). When we pretreated the MCF-10A cells with the PPAR-*γ* antagonist GW9662, we see a reduction in cell motility (Figure 5(a)). ArA treatment increases uPA activity on the cell surface though GW9662 did not seem to fully reduce uPA activity (Figure 5(b)). Additionally, treatment with the *ω*-3 fatty acid DhA, up to 10 *μ*M, had no effect on either cell motility or cell viability (data not shown). These results suggest ArA is able to activate PPAR-*γ*, resulting in increased cell motility and uPA activity.

## 4. Discussion

PAI-1 and uPA protein expression have been used as strong independent prognostic indicators for breast cancer [5, 28–30]. In addition to cancer, PAI-1 overexpression is linked to a variety of disease states. Morbidly obese individuals have elevated circulating PAI-1 levels, likely due to an increase in PAI-1 expression from adipose tissue [31]. In rats with streptozocin-induced diabetes, PAI-1 levels are increased 60–80% over control [32]. In humans, elevated PAI-1 levels have been reported in patients with T2DM [33] and is related to cardiovascular dysfunction [33, 34]. While the literature on PPAR-*γ* activation and PAI-1 alterations is conflicting, it has been shown in a number of cell types and *in vivo* that PPAR-*γ* does modulate PAI-1 expression [34–37]. We treated cells with ciglitazone, 15d-PGJ2, and ArA acid to investigate effects of PPAR-*γ* activation on migration and PAI-1 expression following treatment. Based on previous literature, we expected to see differential effects of PPAR-*γ* activation, specifically with ArA treatment [21].

*In vitro*, treatment of tumor cells with TZDs results in a number of antitumor effects. In prostate cancer cells, PPAR-*γ* ligands reduced proliferation, induced terminal differentiation, and downregulated E-cadherin and c-myc expression [38]. Pioglitazone, in combination with valproic acid, upregulates E-cadherin and reduced invasion and migration in prostate cancer cells [39]. We found that treatment with either ciglitazone or 15d-PGJ2 resulted in a significant decrease in wound closure of MCF-10A cells. Ciglitazone treatment decreased chemotaxis toward EGF in both MCF-10A and MCF-10CA1 cells. GW9662 is a specific PPAR-*γ* antagonist, which binds PPAR-*γ* and blocks ligand binding and subsequent activation of the receptor [40]. Surprisingly, pretreatment with GW9662 did not reverse the effects of ciglitazone, which suggests that ciglitazone mediates this reduction in migration through a PPAR-*γ*-independent mechanism. Emery et al. showed rosiglitazone and pioglitazone inhibited proliferation of pituitary tumors; however, PPAR-*γ* antagonists did not reverse these effects, suggesting the antiproliferative effect was independent of PPAR-*γ* activation [41]. Another study found ciglitazone and 15d-PGJ2 induced apoptosis in normal and malignant B cell, independent of PPAR-*γ* [42]. Finally, ciglitazone and 15d-PGJ2 have been shown to activate p38 MAPK signaling, which were reported to be independent of PPAR-*γ* activation [43, 44].

Interestingly, ArA treatment of MCF-10A cells enhanced cell migration. These effects were reversed in cells pretreated with GW9662, suggesting ArA is acting in a PPAR-*γ*-dependent manner. Since *ω*-3 and *ω*-6 fatty acids have been shown to have differential effects on PPAR-*γ* activation [21], we also investigated if *ω*-3 fatty acids had an effect on cell migration in our system. We saw no change in migration in MCF-10A cells treated with DhA, which agrees with past studies that *ω*-6, but not *ω*-3, fatty acids promote cell motility [45]. GW9662 pretreatment did not fully reverse ArA-induced uPA activity; one possibility for this is ArA also signals through PI3K [46] to upregulate uPA expression [47]. It is also possible ArA is engaging PPAR-*γ* intracellularly, resulting in increased cell migration, while independently initiating the PI3K signaling cascade and then upregulating uPA activity. One limitation of our study was the exclusive use of GW9662 for its irreversible PPAR-*γ* antagonist effect [26, 27]. Future studies with MCF-10A and MCF-10CA1 cells would benefit from either silencing PPAR-*γ* expression or expressing a dominant negative PPAR-*γ* to investigate any possible differences in cell motility or proliferation following treatment with ciglitazone or other PPAR-*γ* agonists. Another limitation to our study was the absence of reporter studies for PPAR-*γ* gene regulation.

TZDs may be useful adjuvant therapies in cancer treatment. One clinical trial in phasetwo investigated the effect of pioglitazone in conjunction with a COX-2 inhibitor in glioma patients and saw moderate results in patients with high-grade glioma, suggesting pioglitazone treatment may be beneficial to a subset of patients [48]. A phase-I trial of a non-TZD PPAR-*γ* agonist LY29311 studied maximum tolerated dose in a combination regimen in patients with advanced solid tumors and determined there was no limiting toxicity and no disease progression [49]. To date, these advances have not been realized with PPAR-*γ* agonists in contrast to their preventative benefits in diabetic patients.

## 5. Conclusions

This study shows ciglitazone treatment reduces both normal and malignant epithelial cell migration *in vitro*, independently of PPAR-*γ* activation. Additionally, we found ciglitazone treatment reduces PAI-1 protein levels, and this effect was not reversed by antagonism of PPAR-*γ*. We hypothesize that the antimigratory effects of ciglitazone are mediated by the alteration of the PA system in these cells. We know PAI-1 inhibits apoptosis, can promote cell motility, and plays a role in intracellular signaling [1, 2]. Given the role of PAI-1 in these tumor processes, the *in vivo* data showing FDA-approved TZDs decrease PAI-1 in diabetic patients, and our results and those of others, one could draw the conclusion that TZD therapies may eventually prove to be a valid adjuvant therapy for some breast cancer patients.
